# Pathogenesis of gallbladder adenomyomatosis and its relationship with early-stage gallbladder carcinoma: an overview

**DOI:** 10.1590/1414-431X20187411

**Published:** 2018-05-21

**Authors:** Liwei Pang, Yan Zhang, Yuwen Wang, Jing Kong

**Affiliations:** 1 Department of Biliary and Minimally Invasive Surgery, China Medical University Shengjing Hospital Shenyang, Liaoning, China; 2 Department of Surgery, The Sixth People's Hospital of Shenyang, Liaoning, China

**Keywords:** Gallbladder adenomyomatosis, Carcinoma, Rokitansky-Aschoff sinuses, Review

## Abstract

The exact pathogenesis of gallbladder adenomyomatosis is still lacking and some controversies over its diagnosis and treatment exist. Originally recognized as a precancerous lesion, adenomyomatosis is currently recognized by recent studies as a benign alteration of the gallbladder that is often associated with cholecystitis and cholecystolithiasis. Gallbladder carcinoma is an extremely malignant disease with a 5-year survival rate of less than 5%. Therefore, it is important to diagnose, differentiate, and confirm the relationship between adenomyomatosis and early-stage gallbladder carcinoma. However, the early clinical symptoms of adenomyomatosis are extremely similar to those of gallbladder stones and cholecystitis, increasing the difficulty to identify and treat this disease. This article summarizes the research progress on gallbladder adenomyomatosis, aiming to improve the understanding of the pathogenesis of adenomyomatosis and further provide insight for its clinical diagnosis and treatment.

## Introduction

Gallbladder adenomyomatosis (GA) is a disease characterized by epithelial proliferation and hypertrophy of the muscles of the gallbladder wall (1) with an outpouching of the mucosa into or through the thickened muscular layer, i.e., the Rokitansky-Aschoff sinuses (RAS) (2). Aldridge et al. first reported that GA was a precancerous lesion (3). Afterwards, several articles suggested that gallbladder cancer originated from adenomyomatosis and the segmental-type adenomyomatosis had a higher incidence rate (4,5). Recently, scholars in China and abroad have suggested that adenomyomatosis in the initial stage was unrelated with gallbladder carcinoma in that it was only proliferation and found in 2.8–5% of all cholecystectomies in China and in 2–5% worldwide (5–7). However, gallbladder carcinoma (GC) is one of the most lethal carcinomas and its treatment continues to be challenging (8). The outcome of GA is poor, and its overall 5-year survival rate is less than 5% (8).

The early symptoms of GC and GA are usually non-specific and the patients only present right upper quadrant abdominal pain. Unfortunately, GC and GA are often associated with gallstones and cholecystitis, which can justify the importance to clarify whether adenomyomatosis has malignant potential or not.

However, the exact mechanism of GA is not fully understood and its relationship with early-stage GC remains unclear. Thus, we aimed to investigate the pathogenesis of GA and its relationship with early-stage GC.

## Types of Gallbladder Adenomyomatosis

GA has three morphological types according to the localization in the gallbladder wall (9): i) fundal (localized) type, ii) segmental type, and iii) diffuse type (10). The fundal (localized) type presents with local thickening, is the most common type and is localized on the fundus of the gallbladder. The segmental type is often found in the body of gallbladder, the lesion is annular and separates the two compartments of the gallbladder. The diffuse type presents with thickening in the unsmooth gallbladder wall (6,11).

## Pathogenesis of Gallbladder Adenomyomatosis

Narrowing of the distal choledoch, neurogenic dysfunction, neuromuscular hyperactivity from abnormal nerve structure, etc. are attributed to restricted bile excretion and increased pressure in the gallbladder, followed by an outpouching of the mucosa into or through the thickened muscular layer, which is called the Rokitansky-Aschoff sinuses (RAS) (12). The hyperplasia of the mucosa and muscle layer of the gallbladder results in wall thickening, decreasing in size, and rising pressure, which can be revealed on cholecystography, especially after fat saturation.

### Insufficiency of gallbladder bud in embryonic period

Some researchers consider that GA may be associated with the insufficiency of gallbladder bud in the embryonic period (13,14). Recently, the first case of GA in an infant was reported (15). Another report has also shown the occurrence of GA in childhood (16), however, there was insufficient evidence to confirm the disease.

### Gallstones and cholecystitis

The long-term stimulation of gallstones and cholecystitis leads to epithelial proliferation and hypertrophy of the muscles of the gallbladder wall. Besides, this stimulation also results in the narrowing of distal choledoch and chronic infection (especially by *Helicobacter pylori*) (17), which inhibits gallbladder activity and increase gallbladder intramural pressure. As biliary stasis occurs in the segmental type, the co-existence with cholelithiasis is the highest at 88.9%, which has been reported to be 47.4% in the fundal and diffuse types (18). Taking into consideration that gallstones and chronic infection (especially by *Salmonella typhi*) are high-risk factors for GC, some scholars suggest that GA should be considered a precancerous lesion of GC (18–20). However, we believe that adenomyomatosis itself cannot cause cancer and that GA and cholecystitis should be seen as carcinogenic factors.

### Hormones

Females are more predisposed than males to gallbladder diseases, including gallstones, GA, and even GC. GA is frequently found in women over 60 years old and estrogen plays an important role. Estrogen can not only increase the rate-limiting enzyme in cholesterol synthesis and HMG CoA reductase activity but also increase cholesterol in the gallbladder, which inhibits the contractile activity of the gallbladder. Moreover, estrogen induces the growth of cholesterol crystals and precipitate stones, and thus may even cause GC (20,21).

### Genes

It is known that Ki67, P53 (11,22), EGFR (23), survivin, and PCNA are related to gallbladder cancer (24). Aldridge et al. (3), Ootani et al. (19), and Nishimura et al. (18) agree that GA is a precancerous lesion. However, Chinese scholars (7,25) find that there is no difference between the expression of Ki67, P53, EGFR, survivin, and PCNA in GA and in chronic cholecystitis. In addition, an article (26) has reported that DNA in the mucosa of GA is similar to that in normal mucosa, suggesting that GA contains no oncogene. Thus, there is insufficient evidence to prove that GA is a precancerous lesion from the perspective of genes.

### Others

Jacobs et al. (27) proposed that GA was related with cholecystocele. Kainuma et al. (14) reported a case of gallbladder adenomyomatosis with pancreaticobiliary maljunction and GA was believed to result from chronic stimulation as an outcome of pancreatic juice reflux. Seok et al. (28) mentioned that heterotopic pancreas of the gallbladder was associated with segmental GA, demonstrating that an ectopic pancreas in the gallbladder may be a risk factor for developing gallbladder cancer. Bedirli et al. (29) proposed that Mirizzi syndrome was a result of chronic inflammation caused by adenomyomatosis.

## Imaging Diagnosis of GA and Carcinoma and their Relationship

Gallbladder cancer is one of the most lethal cancers, surgical treatment is challenging, and the 5-year survival rate is 5–15% (20). Most early-stage gallbladder carcinomas remain asymptomatic, similar to GA, making it difficult to distinguish between them. However, the widespread use of laparoscopic cholecystectomy shows that there is an increase in the number of patients found with incidentally discovered gallbladder cancer (IGBC). Thus, can GA be a precancerous lesion of gallbladder cancer?

In 1991, Aldridge et al. first pointed out that GA was a precancerous lesion (3). After that, Ootani et al. (19) found that 6.4% of 188 segmental type GA were gallbladder carcinoma while about 3.1% developed to be malignant without causing adenomyomatosis, after analyzing 279 specimens. Nabatame et al. (4) also reported rates of 6.6% (22/334) and 4.3% (181/4226) for the above conditions. Recently, Nishimura et al. (18) suggested that adenomyomatosis was a precancerous lesion. Kai et al. (30) speculated that gross appearance of GA precedes carcinogenesis. According to these opinions, segmental type GA has a higher risk of developing into gallbladder cancer to some extent. Other types of GA, including fundal and diffuse types, do not show a strong association with malignancies of the gallbladder.

However, some scholars believe that adenomyomatosis is unrelated to gallbladder carcinoma and they reached such a conclusion by comparing the expression of EGFR, P53, survivin, Rb, and cyclinD1 of GA to that of gallbladder cancer (7,25,31). None of the 113 GA patients among 4704 consecutive patients developed cancer (11). Based on histology, adenomyomatosis is an epithelial proliferation and hypertrophy of the muscles of the wall while early-stage gallbladder cancer presents cell dysplasia (32,33). Therefore, we cannot be certain that gallbladder adenomyomatosis is a precancerous lesion.

Although GA is a benign epithelial proliferation, other factors secondary to GA, such as stones, cholecystitis among others may lead to cancer and dysplastic changes. Because the relationship between GA and GC is not clear determines that the differentiation between early-stage GC and GA be difficult. Ultrasound (US) is the exam of first choice for diagnosing gallbladder disease, but this method has low accuracy and depends largely on the operator’s skills. The observed features of GA on US are: a) tiny anechoic cystic spaces (i.e. Rokitansky-Aschoff sinuses); b) focal or diffuse gallbladder wall thickening; c) intramural echogenic foci; and d) twinkling artifact (also called comet-tail), which is one of the major differential diagnoses between GA and GC (34–37). As for GC, the US has low accuracy, reaching 22% accuracy in early stages (35), and it cannot distinguish between GC and chronic cholecystitis (16). As for computed tomography (CT), a characteristic of GA is a rosary sign, showing mucosal epithelium with intramural diverticula (31,33,36) and indicates a suspicion of cancer after US. On magnetic resonance imaging (MRI), RAS present with small, rounded, high signal intensity foci, called “pearl necklace sign” (38), especially after fat saturation. The most accurate examination is high-resolution ultrasound (HRUS), which can help distinguish GA from early-stage wall-thickening-type GC. The findings on HRUS are the definite presence of intramural cysts (RAS), intramural echogenic spots, and cholesterol deposits in the RAS (36). As for the receiver operating characteristic (ROC) curve (Az), a report (35) showed that HRUS had high Az values (greater than 0.90) for differentiating GA from early-stage, wall-thickening-type GC through symmetrical wall thickening, intramural cystic spaces, intramural echogenic foci, and twinkling artefacts, which were significantly associated with GA. However, irregular thickening of the outer wall, focal innermost hyperechoic layer (IHL) discontinuity, IHL thickening greater than 1mm, loss of multilayer pattern in the gallbladder wall, and intralesional vascularity were significantly associated with GC. In recent years, Bonatt et al. (39) summarized imaging findings of GA, and concluded that US represents the imaging modality of choice for diagnosing GA and MRI is used for unclear cases after US. We believe that HRUS is the best choice to distinguish GA from early-stage wall-thickening-type GC.

## Conclusions

GA cannot be regarded as a precancerous lesion based on available evidence. GA is a benign epithelial proliferation while stones and cholecystitis secondary to GA may lead to dysplastic changes and cancer. Most patients with GA do not have typical symptoms and usually present chronic cholecystitis and vague abdominal pain, which complement histological examination of cholecystectomy specimens (40–43). The increasing gallbladder intramural pressure may indicate that gallbladder decompression delays the adenomyomatosis progress. Although we have contributed to a further understanding of the causes of GA, its current pathogenesis is still not fully understood. With the development of imaging technology, the detection rate of RAS can improve and increase the diagnostic rate of GA before operation. However, due to limited imaging equipment and technical difficulties, the possibility of misdiagnosis is large.

At present, the main treatment for GA is surgery, but it is very important to screen patients and make clear the operation indications, taking the complications after cholecystectomy into consideration. As for asymptomatic GA, a conservative treatment is recommended with US exams twice a year. The fundal type GA can be treated by partial laparoscopic cholecystectomy. The segmental and diffuse type should undergo a total laparoscopic cholecystectomy. Females over 60 years of age who present gallbladder stones and segmental type GA should undergo surgery (4,44–46).
